# Identifying Eating Occasion-Based Opportunities to Improve the Overall Diets of Australian Adolescents

**DOI:** 10.3390/nu9060608

**Published:** 2017-06-14

**Authors:** Flavia Fayet-Moore, Andrew McConnell, Jean Kim, Kevin C. Mathias

**Affiliations:** 1 Nutrition Research Australia, Sydney, NSW 2000, Australia; 2 Nestlé Australia Ltd., Rhodes, NSW 2138, Australia; 3 Nestlé Research Center, 1000 Lausanne, Switzerland

**Keywords:** adolescents, food intake, nutrient intake, eating occasions, dietary quality, meal patterns, shortfall nutrients, discretionary energy, Australian diets, food-based recommendations

## Abstract

Adolescents in Australia have a poor dietary intake, leading to large numbers of them being at risk for inadequate intake of micronutrients, and excessive intake of less healthful dietary components. This study examined dietary intakes at multiple eating occasions to identify opportunities for more targeted recommendations and strategies to improve dietary intakes among adolescents. Data from the first 24-h recall of 14–18 years old in the 2011–2012 National Nutrition and Physical Activity Survey were analysed (*n* = 772). Participant-defined eating occasions were classified as breakfast, lunch, dinner or other eating occasions combined. The mean percent contribution to the total day intake of top shortfall nutrients (calcium, magnesium, vitamin A, iron), discretionary calories, saturated fat, free sugars and sodium, as well as nutrient density, the foods consumed and the percent of consumers at each eating occasion, were calculated. Breakfast had the lowest prevalence of consumers (81%), contributed the least to total daily energy (14.6%) and almost a quarter of daily calcium and iron. Other eating occasions combined contributed 47.5% of free sugars and were top contributors of daily calcium (34.6%) and magnesium (31.7%). Discretionary foods contributed 32.4% of the energy at lunch, and the sodium content at lunch was 415 mg/1000 kJ. Key opportunities identified for adolescents were to increase breakfast consumption, given the high nutrient densities of breakfasts consumed; improve overall lunch quality, particularly the sodium content; promote the intake of milk, fruit and a variety of vegetables at both lunch and dinner; maintain healthful choices at in-between meal eating occasions while focusing on decreasing the intake of discretionary foods.

## 1. Introduction

Adequate nutrition throughout adolescence is important for ensuring optimal growth, development and health [1,2,3]. The nutritional requirements of adolescents increase markedly from childhood, with comparatively higher requirements for almost all macro- and micronutrients [4]. Despite the importance of adolescent nutrition, large percentages of 14–18 years old are at risk for inadequate intakes of calcium (90% of girls, 71% of boys), magnesium (72% of girls, 61% of boys), iron (40% of girls, 8% of boys), and vitamin A (27% of girls, 33% of boys) [5]. The quality of the diet in this age group is also poor, with low intakes of fruits, vegetables, dairy, and lean protein sources, as well as excessive intakes of sodium, free sugars and the highest proportion of energy from discretionary foods (41%)—foods not necessary for a healthy diet—among all age groups in Australia [6,7,8]. Examining the dietary intakes of the multiple eating occasions that comprise the total daily food and nutrient intakes may identify new opportunities for more targeted recommendations and strategies to help improve the dietary intakes of adolescents.

Previous analyses of individual eating occasions have provided insights for improving overall dietary intake. Breakfast consumption has long been associated with higher diet quality in adolescence, with breakfast consumers consistently having higher intakes of several key nutrients compared to those that skip breakfast [9,10]. A cross-sectional study of European adolescents also found that the nutritional quality of breakfast tends to worsen with increasing age. Lunch consumption has also been associated with higher micronutrient intakes among adolescents in the US, but differences between intakes of discretionary energy (added sugars and solid fats) were not shown between lunch consumers and lunch skippers [11]. While it has been previously reported that almost all adolescents in Australia consume dinner [12], the type of evening meal has been shown to be associated with total diet quality. Consumption of commercially prepared meals was associated with a lower Dietary Guideline Index score in young Australian adults [13].

Inherently, examining individual eating occasions in the context of the total diet does not provide a complete perspective on the impact that each eating occasion has on total daily intake, and subsequently, the potential opportunities to improve overall dietary quality. Examining multiple components of each eating occasion (e.g., percentage of consumers, nutrient density, and foods and beverages consumed) can further identify more specific areas to target to improve dietary intake. Therefore, the aim of this study was to take a holistic approach to investigate how food and nutrient intakes at multiple eating occasions contribute to the total daily intake of Australian adolescents. It is not expected that all nutrients and foods should be equally distributed across all eating occasions; however, by examining how each eating occasion contributes to the total daily diet, new opportunities to improve dietary intakes of adolescents in Australia may be identified. Specifically, we aimed to assess the intake of the top shortfall nutrients (calcium, magnesium, iron and vitamin A) as well as the top foods and nutrients recommended by the Dietary Guidelines to limit (saturated fat, free sugars, sodium and total discretionary food intake) by eating occasion.

## 2. Materials and Methods

### 2.1. Survey Methodology

Data from the 2011–2012 National Nutrition and Physical Activity Survey (NNPAS) were used. The NNPAS was a nationally representative survey conducted by the Australian Bureau of Statistics (ABS) as part of the 2011–2013 Australian Health Survey [14]. The Australian Health Survey used a stratified multi-stage area sampling plan to identify private households across urban and rural dwellings in all states and territories in Australia. Within each household, one adult and one child were randomly selected; interviews were conducted throughout the year to account for seasonal variation in intakes. Data were collected with parents present during the interview for 12–14 years old, and independently for those aged 15 years and older. The NNPAS collected detailed dietary information in a face-to-face 24-h recall by trained interviewers using the Automated Multiple-Pass Method developed by the Agricultural Research Service of the United States Department of Agriculture [15], to capture all foods and beverages consumed by respondents the day prior to the interview day. A second day of dietary recall was provided when possible, via telephone interview, by approximately two-thirds of respondents. In order to maximise adolescent sample size, the first day of recall was utilised for this study and thus, data from the 774 respondents aged 14–18 years were examined. Dietary intake data were analysed using the survey specific 2011–2013 Australian Food Composition Database (AUSNUT) developed by Food Standards Australia New Zealand [16]. The interview components of the NNPAS were conducted under the Census and Statistics Act 1905 and ethics approval was not necessary.

### 2.2. Eating Occasions

As part of the 24-h dietary recall, respondents were asked to classify the eating occasion (EO) for each food and beverage consumed from 11 pre-defined options: breakfast, brunch, morning tea, lunch, afternoon tea, dinner, supper, snack, beverage/drink, extended consumption and other. For the purposes of these analyses, lunch and brunch were recoded as lunch, breakfast and dinner remained the same, and the remaining eating occasions were grouped as ‘other eating occasions combined’. The percentage of respondents who reported a food or beverage at each EO was reported as the percentage of consumers.

Respondents were not restricted to reporting one eating occasion at a given time, and hence could classify a food such as muesli as ‘breakfast’ at 8:00 and juice as ‘beverage/drink’ also at 8:00. We investigated how often two different eating occasions were reported at the same time of day. There were 100 instances of ‘other eating occasions combined’ occurring at the same time as breakfast, lunch, or dinner and all of these occasions were beverages (90 were water) categorised as ‘extended consumption’. The ‘extended consumption’ eating occasion captured foods where the total amount across the day was reported, but not necessarily how much was consumed periodically, throughout the day. Hence it was decided that these 100 foods should remain as ‘other eating occasions combined’ and not be coded as breakfast, lunch, or dinner.

### 2.3. Dietary Intake Data

Discretionary foods are defined by the Australian Dietary Guidelines as foods and beverages not necessary for a healthy diet and that are generally “high in saturated fat and/or added sugars, added salt or alcohol and low in fibre” [17]. The foods and beverages in the 24-h recall were defined as discretionary or non-discretionary based on coding provided by the ABS. Free sugars are defined by Food Standards Australia New Zealand as all added sugars (added forms of dextrose, fructose, sucrose, lactose, sugar syrups, fruit syrups), as well as the sugars in honey, fruit juice, and fruit juice concentrates [18].

The per capita percent contribution to total daily intake for energy and nutrients was calculated for each eating occasion. Two approaches were used to calculate nutrient density among consumers of the eating occasion. First, the mean nutrient intake per kilojoule consumed at each eating occasion was calculated. For nutrients that contribute energy (saturated fat, discretionary energy and free sugars) the density was calculated as the percentage of energy at the eating occasion contributed by the nutrient. For example, the discretionary energy intake (kJ) at lunch was divided by the energy intake (kJ) at lunch, and then multiplied by 100. For the micronutrients, nutrient density was calculated per 1000 kJ. Given the different units for each nutrient, the second approach created a nutrient density index to facilitate examining all nutrients simultaneously within each eating occasion. For a given eating occasion, the percentage of total daily nutrient intake was divided by the percentage of total daily energy intake to give a nutrient density relative to the total day, Equation (1). This nutrient density index is algebraically equivalent to dividing the nutrient density of an eating occasion, by the nutrient density of the total day among consumers of a particular eating occasion, Equation (2).

(1) $$\frac{Calcium_{breakfast}/Calcium_{total}}{Energy_{breakfast}/Energy_{total}}=\frac{\%\;of\;daily\;Calcium}{\%\;of\;daily\;Energy}$$

(2) $$\frac{Calcium_{breakfast}/Calcium_{total}}{Energy_{breakfast}/Energy_{total}}=\frac{Calcium_{breakfast}/Energy_{breakfast}}{Calcium_{total}/Energy_{total}}$$

The comparison of the nutrient density index of a single eating occasion to the total day, provides context beyond the first approach, where nutrient densities are reported as nutrient/energy (kJ). An index of 1 occurs when the nutrient density of a given eating occasion is equal to the nutrient density for the total day among consumers of that eating occasion, Equation (2). It is important to note that given the low quality of the total day, nutrient density indexes above 1 for micronutrients, and nutrient density indexes less than 1 for nutrients to limit, do not necessarily indicate a lack of need to make improvements.

### 2.4. Food Groups

In this study, the sub-major food group classification was utilised to rank food groups as top contributors to total daily energy and nutrients. There were 132 sub-major food groups in the 2011–2013 AUSNUT database [16]. The top five sub-major food groups that contributed the most to total daily energy, discretionary energy, saturated fat, free sugars, sodium, calcium, magnesium, iron and vitamin A were ranked, and the sum of the percent contribution of the top five sources was calculated. The percent contribution to the total day for the top five sub-major food groups was calculated for each eating occasion, to allow identification of the top opportunities to decrease or increase total daily intake.

The food group ‘mixed dishes where cereal is the major ingredient’ covers a wide range of foods including burgers, pizzas, sandwiches, and pasta and rice dishes. Therefore, this sub-major food group was further sub-divided into the groups: burgers, pizza, tacos, sandwiches, pasta/dumplings, and rice dishes/sushi. In order to profile dietary intake patterns of adolescents, the 132 sub-major food groups were further aggregated using both sub-major and minor food groups into 52 food groups (Supplementary Table S1). The proportion of respondents who consumed the food group at each eating occasion (Supplementary Table S2) was calculated, as well as the mean grams consumed for the following food groups: commercial fruit/dried fruit, fruit, fruit and vegetable juice, potatoes, tea and coffee, vegetables (non-potato), and water; and also the mean energy intake per capita for all food groups (Supplementary Table S3).

### 2.5. Statistical Analyses

Analyses were performed using IBM SPSS, version 23.0 (IBM Corp., Armonk, NY, USA). Data were weighted to be representative of the Australian population using weighting factors provided by the ABS. To be consistent with similar results reported by ABS, the margin of error was calculated at the 95% confidence level for the proportion of energy and nutrients coming from each food group. The standard error of the mean was calculated for energy and nutrient intakes from eating occasions and food groups, percent energy and nutrient contributions, and nutrient densities and density indexes.

## 3. Results

### 3.1. Energy and Nutrients at Each Eating Occasion

#### 3.1.1. Breakfast

Breakfast had the lowest prevalence of consumers (81%), and contributed the least to total daily energy (14.6%) of all eating occasions (Table 1). Breakfast contributed a lower proportion of total discretionary energy (8.3%), saturated fat, and sodium than its energy contribution, but a higher proportion of total free sugars (16.3%). Subsequently, the nutrient density indexes were less than 1 for discretionary energy, saturated fat, and sodium, while free sugars was 1.2. With respect to micronutrients, breakfast contributed almost a quarter of daily calcium and iron, and over 17% of daily magnesium, and vitamin A. Nutrient density indexes at breakfast were all above 1 for calcium, iron, vitamin A, and magnesium.

#### 3.1.2. Lunch

The prevalence of lunch consumers was 87.6%, and lunch contributed just under a quarter (22.9%) of total daily energy (Table 1). Relative to its energy contribution, lunch contributed proportional amounts of discretionary energy (20.6%) and saturated fat (22.1%), but relatively less free sugars (17.3%) and more sodium (29.8%). Discretionary foods contributed 32.4% of the energy at lunch, and the sodium content at lunch was 415 mg/1000 kJ. The nutrient density indexes for discretionary energy, saturated fat and free sugars at lunch were all below 1, while sodium was 1.4. Nutrient density indexes for all micronutrients at lunch were ≤1.

#### 3.1.3. Dinner

Dinner contributed 32.2% of total energy intake, 32.3% of daily sodium and 32.7% of daily saturated fat (Table 1). Nutrient density indexes were all 1 or lower for discretionary energy, saturated fat, free sugars, and sodium. Dinner contributed the highest proportions of daily iron (33.3%) and vitamin A (37.5%). The nutrient density indexes for iron and vitamin A were above 1 and below 1 for calcium and magnesium. Although dinner contributed a third of daily iron, the iron density at dinner was nearly half of the iron density at breakfast.

#### 3.1.4. Other Eating Occasions Combined

Almost all respondents were consumers of at least one, non-main meal eating occasion (99.2%). These occasions contributed 30.2% of total daily energy (Table 1). Other eating occasions combined contributed 48.2% of daily discretionary energy, 47.5% of free sugars, and 33.0% of total saturated fat. Discretionary foods contributed 57.0% of the energy at other eating occasions, saturated fat contributed 13.1%, and free sugars contributed 24.0%. The nutrient density index for sodium was 0.7, and discretionary energy and free sugars were both 1.8. Other eating occasions combined were top contributors of total daily calcium (34.6%) and magnesium intake (31.7%) and both had a nutrient density index of 1.2.

### 3.2. Food Groups and Nutrient Contributions

Examining how eating occasions contribute to the top sources of daily nutrients can identify specific foods and beverages to target at each eating occasion, but also the top opportunities across all eating occasions to improve total daily intakes. Examining the percent contribution of foods and beverages to the total day provides context for recommendations to change total daily intakes via a single source of a nutrient at a given eating occasion.

#### 3.2.1. Breakfast

At breakfast, fruit and vegetable juices and drinks contributed 4.2%, and sugars, honey, and syrups contributed 3.4% to daily free sugars (Table 2). Dairy milk was among the top five contributors to daily calcium, vitamin A, and magnesium. Dairy milk consumed at breakfast contributed 10.8% of total calcium, 4.5% of total vitamin A, and 3.0% of total magnesium. Ready-to-eat breakfast cereal was the top source of daily iron intake and the second top source for magnesium. Ready-to-eat breakfast cereals consumed at breakfast contributed 15.6% of total daily iron and 5.7% of total daily magnesium.

#### 3.2.2. Lunch

Three of the top five food groups that contributed the most to total daily sodium were also top contributors at lunch (Table 2). At lunch, breads and rolls contributed 4.9%, burgers 2.8%, pastries 2.4%, and processed meat contributed 2.2% of daily sodium intakes. Breads and bread rolls consumed at lunch contributed 4.0% of daily iron intake and 3.5% of total daily magnesium intake. While dairy milk was a top five source of daily calcium and vitamin A, the calcium (0.5%) and vitamin A (0.2%) from dairy milk at lunch was the lowest of any eating occasion, whereas cheese consumed at lunch contributed 3.8% to daily calcium. Soft drinks and flavoured mineral waters were a top contributor to the total daily intake of free sugars (6.3%) and the top contributor of free sugars at lunch. Carrot and similar root vegetables contributed the most to total daily vitamin A. While carrots and similar root vegetables contributed 3.7% of the daily vitamin A at lunch, at dinner, they contributed nearly three times more (12.1%).

#### 3.2.3. Dinner

Burgers and pizza were among the top five contributors to daily sodium intake, and dinner is a top opportunity to address sodium consumption from these two foods groups. Pizza and cheese consumed at dinner contributed 3.4% and 2.4% of daily calcium, respectively. Beef, sheep, and pork consumed at dinner were a top contributor to daily iron (4.6%), which was nearly three times lower than the contribution of ready-to-eat breakfast cereals consumed at breakfast (15.6%). Soft drinks and flavoured mineral waters were a top contributor to daily free sugars (7.2%) and a top contributor at dinner. Carrots and similar root vegetables consumed at dinner contributed the most to daily vitamin A intakes (12.1%). Consumption of potatoes at dinner contributed 2.8% to daily magnesium intake, but consumption of the other top daily sources of magnesium (bread and bread rolls, ready-to-eat breakfast cereals, waters, and dairy milk) contributed less than 1%.

#### 3.2.4. Other Eating Occasions Combined

Soft drinks and flavoured mineral waters; cakes, muffins and cake-type desserts; and chocolate and chocolate-based confectionery were among the top five contributors to daily intake of discretionary energy, including free sugars. Limiting consumption of these at other eating occasions is a top opportunity to address intake of these foods groups (Table 2). Chocolate and chocolate-based confectionery was among the top five sources of saturated fat across the day, and contributed 4.5% of daily saturated fat at other eating occasions combined. Dairy milk, and flavoured milk and milkshakes consumed at other eating occasions combined, contributed 8.7% and 3.7% of daily calcium, respectively. Dairy milk, frozen milk products, and flavoured milk and milkshakes consumed at other eating occasions combined contributed 3.6%, 3.0%, and 2.5% of daily vitamin A intake, respectively. Waters was the third highest contributor to daily magnesium intake and its contribution was nearly exclusively from other eating occasions combined (5.5%). While some foods and beverages may be discretionary and also contain micronutrients (e.g., ice-cream and milkshakes), the top five sources of daily calcium and magnesium were distinct from the top five sources of discretionary energy.

### 3.3. Percent Consumers of Aggregated Food Groups

Prevalence of consumption of the aggregated food groups by eating occasion gives an indication of why certain food groups were low or high sources of nutrients among adolescents’ diets (Supplementary Table S2). Consumption of sweetened beverages among adolescents was distributed across all eating occasions, with the lowest prevalence at breakfast (6.7%) and the highest at other eating occasions combined (23%). Milk consumption was highest at breakfast (40%) and other eating occasions combined (24%), with only 5% consuming at dinner and 3% at lunch. Lunch had the highest percentage of consumers of breads and bread rolls (34%), savoury pastries (7%), and processed meat (14%). Fruit was predominantly consumed at other eating occasions combined (36%), followed by 12% at lunch, 6% at breakfast, and 3% at dinner. Nut and seed product consumption was very low (≤5% for each eating occasion). Potatoes and vegetables were consumed primarily at main meals: 24% consumed potatoes at dinner and 10% at lunch; and 27% consumed vegetables at dinner and 17% at lunch. Consumption of savoury snacks, chocolate and confectionery, and desserts were primarily at other eating occasions combined: 17% had savoury snacks, 26% had chocolate and confectionery, and 41% had desserts.

## 4. Discussion

Given the large percentages of adolescents in Australia at risk for inadequate intakes of micronutrients and excessive intakes of less healthful dietary components, this study utilised a holistic dietary analysis approach, by examining all eating occasions across the day, to determine if the whole diet is of poor quality, or just certain eating occasions. For each eating occasion, examining the prevalence of consumers, the quality of the eating occasion (nutrient density) and the top foods and beverages consumed, provided insights into why certain eating occasions contributed larger or smaller amounts of nutrients, and identified potential opportunities to improve dietary intake among Australian adolescents. The key opportunities identified were to develop strategies to increase breakfast consumption, given the high quality of breakfasts consumed; improve the nutrient quality of lunch, particularly the sodium content; promote the consumption of dairy milk, fruit, and a greater variety of vegetables at both lunch and dinner; a focus on decreasing sweetened beverage consumption at lunch, dinner and at other eating occasions; and promoting healthy foods and beverages at other eating occasions, or non-main meal eating occasions, with the encouragement of fruit and dairy milk intake, while focusing on strategies that decrease consumption of discretionary foods and beverages, including those that are the top sources of free sugars.

While breakfast contributed the least number of kilojoules to the total day (15%), partially due to having the greatest prevalence of skippers, breakfast contributed a relatively larger proportion of all shortfall nutrients for its energy contribution. The high micronutrient density and low contribution from discretionary energy and sodium to the total day at breakfast, provides an opportunity to promote breakfast consumption among adolescents in Australia. These findings are consistent with previous studies that showed that breakfast consumption is associated with higher daily nutrient intakes among children and adolescents [9,19,20]. A recent systematic literature review of meal skipping studies in young adults found a lack of time to be consistently the primary reason given for meal skipping [21]. This is comparable with research that includes Australian adolescents, that found a lack of time and not being hungry in the morning as the most common reasons for skipping breakfast [22,23]. There have been a number of intervention studies designed to increase breakfast consumption, with mixed results. A 2011 systematic review of 11 intervention studies, almost all of which were done on children and adolescents, found that only three of these studies resulted in an increase in breakfast consumption at follow-up [24]. No study on food provision alone was found to increase breakfast consumption frequency, but food provision has been shown to improve breakfast quality, and the frequency increased when it was promoted with persuasive messages about the health benefits of breakfast. This finding is consistent with research that food scarcity is not a common reason for breakfast skipping [25], and with research that suggests positive attitudes about breakfast consumption are associated with actual consumption [26]. In Australia, a large-scale national breakfast promotion campaign was associated with reduced rates of breakfast skipping in Australian school students [27]. A systematic literature review of family correlates and breakfast consumption in children and adolescents found that parental breakfast eating and living in two-parent families were the most consistent factors associated with breakfast consumption [28]. The evidence suggests that a family-focused nutrition education program is potentially the most effective method for modifying breakfast frequency among adolescents.

The quality of the lunch meal presents an opportunity to improve adolescents’ lunch intakes by increasing calcium, iron, magnesium, and vitamin A, while decreasing discretionary foods and beverages, particularly those high in sodium. Lunch was the second most skipped meal in this study. Studies in the US have shown that skipping lunch is associated with lower daily nutrient intakes [11], and therefore, promoting lunch consumption among adolescents in Australia could be an effective strategy, if the quality of the lunch meal is improved, by focusing on food and beverage choices that have higher contents of the shortfall micronutrients. In Australia, an analysis of the 1995 National Nutrition Survey found that 86% of Australian school children aged 5 to 15 years brought their lunch from home and 40% of their energy intake was consumed during school hours [29]. More recent evidence shows that the canteen is also used by 95% of children to supplement food brought from home [30]. Research indicates that most Australian school lunches contain a sandwich, piece of fruit and multiple snacks [29,31,32], and there appears to be an increase in fruit, packaged snacks, muesli/fruit bars, cakes, and biscuits in the lunch box [32]. Further analysis of both in- and out-of-school lunches is needed to provide insights for strategies to improve dietary intakes at the lunch meal. Lunch may be improved by limiting sweetened beverage access, providing lower sodium food choices, and increasing the amount of vegetables and dairy being consumed. Ongoing campaigns for healthier lunch boxes have been implemented [33] and have shown to be effective at promoting fruit and water in the lunch boxes of primary school children in Australia [34]. Perhaps the ‘lunch box’ in the Australian culture is a limiting factor in nutrient-dense lunches, which inherently limits the type and availability of foods that can be brought from home. Re-heating facilities and access to refrigeration at schools may be considered as a strategy to increase the variety of foods that can be consumed during school and the 2010 National Healthy School Canteen Guidelines has been established to provide national guidance and training to assist canteen managers to encourage healthier food and drink choices at school canteens in Australia [35]. A barrier for initiatives to increase the access to re-heating and refrigeration in schools may be the cost of additional facilities; however, there is a growing body of evidence highlighting substantial economic savings with dietary changes that result in disease risk reduction [36,37,38,39]. The provision of re-heating facilities and access to refrigeration at schools as part of a Government funded program aimed at promoting healthy eating in schools, may even have potential to bring cost savings in the long-term. School meal programs have been shown to improve lunch quality [40,41], and perhaps Australian adolescents would benefit from a national school lunch program, campaigns to improve the contents of packed lunches, or improvements in options available in the canteens.

Given the high proportion of daily energy and low percent of skippers at dinner, nutrient intake at dinner can also be targeted to improve overall intake. One strategy would be to encourage the consumption of a wider variety of vegetables, in addition to the potatoes which were popular. Further, a much greater proportion of adolescents consumed sweetened beverages at dinner (23%) compared to milk (5%). Switching the type of beverage consumed at dinner may help increase calcium intake and potentially decrease intake of sweetened beverages and consequently, free sugars. Given that burgers and pizza were top contributors to calcium and iron intakes, it is necessary to find ways to lower the sodium content of these foods. One approach would be to promote intake of home cooked meals, as commercially prepared meals (e.g., takeaway food, pre-packaged, restaurant) were associated with a poorer diet quality [42]. Again, a family focused approach would be beneficial. Parental presence at the evening meal is positively and consistently associated with the diet quality of adolescents [43,44,45] and the frequency of a family meal during adolescence has been shown to predict dietary quality several years later [46].

Other eating occasions combined contributed a large proportion of daily energy, were top contributors of calcium, magnesium, and vitamin A, and provided discretionary energy, including free sugars. While some food groups (e.g., dairy desserts) might contribute both shortfall nutrients and added sugars, the results showed that the choices at other eating occasions combined were both healthful (e.g., waters, fruit, dairy milk) and less healthful (e.g., sweetened beverages, chocolate and chocolate-based confectionery and desserts). These occasions fall outside main meals and are typically classified as snacking. Snacking has been associated with higher energy, lower fruit and vegetable intake, higher sweetened beverage consumption, and more frequent fast-food intakes in a recent analysis of the diets of U.S. adolescents [47]. In Australia, previous research has shown that adolescents consumed half of their total daily intake of added sugars at non-main meal occasions, which was similarly defined as all eating occasions other than breakfast, lunch and dinner [48]. Intuitively, the act of snacking does not have to be associated with lower quality diets or higher calorie intakes, depending on the type of snacks chosen. Preliminary evidence for this has shown that energy-dense and nutrient-poor snacks were positively associated with body mass index *z*-score, whereas, the total number of snacks consumed per day were inversely related to body mass index *z*-score [47]. Given that other eating occasions combined contributed a significant proportion of daily intakes of shortfall nutrients, specifically calcium, promotion of more healthful choices and focusing on lowering intakes of sweetened beverages, savoury snacks, desserts, and confectionery at eating occasions other than breakfast, lunch and dinner, is warranted.

With respect to daily intakes of specific micronutrients, 40% of adolescent girls and 8% of adolescent boys were at risk for inadequate intakes of iron. While protein intakes were adequate in these subpopulations [5], the top two sources of iron were from plant-based, iron fortified sources (breakfast cereals and breads and bread rolls). With regard to higher iron bioavailability and density, animal-based protein sources may be another strategy to address iron intake, particularly among females, as it has been previously shown to be a less popular food choice among Australian children and adolescents [49] and avoided by young female adults [50,51]. Research also shows that children are more likely to consume vegetables in the evening meal if they also consume meat [52]. Therefore, promoting animal-based sources high in iron at the evening meal, with greater vegetable variety, including vegetables that are also high in iron, may help address low iron intakes and improve diet quality.

Australian adolescents had low intakes of foods and beverages that are particularly high in magnesium, such as nuts, which have been shown among both adolescents and adults in the US to be associated with a lower likelihood of being below the Estimated Average Requirement (EAR) for magnesium [53] and with more nutrient-rich diets [54]. Consumption of nut and seed products among Australian adolescents was 12%, with the highest proportion of consumers at breakfast (5%) followed by lunch (3%). Despite bans on nuts in some schools, the National Healthy School Canteen Guidelines recommends un-salted, un-roasted or dry-roasted nuts as a nutritious food that should be on the canteen menu every day (if your school allows them) [35]. Consumption of dark leafy vegetables, beans and other magnesium-dense foods were not in the top five food group sources, but also present opportunities to increase magnesium intakes at all eating occasions.

There are several strengths and limitations to this study. The use of population means in this study identifies opportunities to improve the dietary quality of the population as a whole, but not necessarily the best strategies for individuals who are at risk for inadequate nutrient intakes and who would benefit the most from increasing the quality of their diets. Given that the micronutrient requirements of individuals are not available, it is not possible to target the behaviours of individuals that are not meeting their shortfall nutrient targets. The large percentages of adolescents at risk for inadequate intakes of shortfall nutrients, and the excessive intake of discretionary energy, including free sugars and sodium, provides confidence that the opportunities identified, if addressed, would benefit both the population as a whole, and those individuals at risk. However, it is possible that some individuals with the greatest needs for dietary improvements would not benefit from some of the strategies derived from this study (e.g., individuals with inadequate intakes of shortfall nutrients that habitually consume a high-quality breakfast and/or lunch). Similarly, due to the breadth of the analysis, we did not examine if the potential strategies identified for the population as a whole differed by other factors, such as sex or socioeconomic status.

Further, the opportunities identified in this study are numerically the top approaches to improve intakes, but not necessarily the most feasible or practical, from a dietary behaviour standpoint. It is possible that some other strategies may be easier to implement and therefore could have a greater impact on dietary quality. Implementation should take into account both the feasibility and potential effectiveness to improve dietary intakes of adolescents in Australia. Due to the self-reported dietary intake data and under-reporting of energy intake among adolescents in the 2011–2012 NNPAS [55], interpretation of absolute values should be made with caution. This analysis focused on relative comparisons across eating occasions and relies on the assumption that miss-reporting was proportionally similar across eating occasions, and that any deviations would not severely bias the population means. One particular concern would be that the micronutrient densities of other eating occasions could be overestimated given the potential for adolescents to under-report less healthful foods and beverages; however, the analyses by food groups confirms that top sources of calcium and magnesium were consumed at other eating occasions.

A strength of this analysis is that multiple strategies were identified at each eating occasion to improve overall dietary intakes, the use of a representative sample of adolescents, the examination of eating occasions across the day, rather than the focus on a single occasion (such as breakfast), and the use of multiple variables (i.e., the prevalence of consumers, nutrient density, nutrient contribution, food groups) provide a full picture of the eating occasion relative to the total day’s intake. Given the relatively small contributions to the total day for individual food and beverage groups, multiple strategies are needed to have a significant impact on the dietary quality of adolescents in Australia. The use of self-reported eating occasions allows for interventions to be communicated using the same terminology as adolescents use to report their food intake, although a more objective measure of meal classification, such as time of day, may have provided different results, particularly with respect to the food groups being consumed. Not only were the top food group sources of the shortfall micronutrients by eating occasion investigated, but the prevalence of consumers of all food groups. This provided context to identify, not only what adolescents were eating, but also what they were not eating. Lastly, due to the complexity of the analysis, it was not possible to identify other eating occasions on their own. These eating occasions such as ‘beverage’ and ‘snack’ could have nutrient densities that are superior to one another, and further analysis of their breakdown may give insights as to which occasions have better nutrient contributions and profiles.

## 5. Conclusions

In conclusion, this study identified eating occasion-specific opportunities with the potential to improve dietary intakes by increasing intake of shortfall nutrients among Australian adolescents. The key opportunities identified were increasing the number of adolescents consuming breakfast; increasing the quality of the lunch meal; promoting a wider variety of, and increasing, vegetable intake at lunch and dinner; increasing dairy milk intake and decreasing sweetened beverage intakes at all eating occasions, with a focus on lunch, dinner and other eating occasions; increasing fruit consumption at lunch and at other eating occasions; increasing intake of magnesium rich foods like nuts; and a focus on improving choices available, and made, outside of main meal eating occasions.

## Figures and Tables

**Table 1:** Energy and nutrient contribution to total daily intake, nutrient density, and nutrient density index by each eating occasion.

Eating Occasion ^1^	Energy	Discretionary Energy	Saturated Fat	Free Sugars	Sodium	Calcium	Iron	Vitamin A	Magnesium
	Contribution to Total Daily Intake (% ^†^ ± SE *)								
**Breakfast**	14.6 ± 0.4	8.3 ± 0.6	12.2 ± 0.5	16.3 ± 0.8	13.5 ± 0.5	23.4 ± 0.8	23.0 ± 0.7	17.1 ± 0.8	18.7 ± 0.5
**Lunch**	22.9 ± 0.6	20.6 ± 1.0	22.1 ± 0.8	17.3 ± 0.9	29.8 ± 0.8	17.8 ± 0.7	21.8 ± 0.6	18.2 ± 0.8	20.0 ± 0.5
**Dinner**	32.2 ± 0.6	22.1 ± 1.0	32.7 ± 0.8	18.5 ± 0.9	32.3 ± 0.8	24.2 ± 0.7	33.3 ± 0.7	37.5 ± 1.0	29.6 ± 0.6
**Other combined**	30.2 ± 0.7	48.2 ± 1.2	33.0 ± 0.9	47.5 ± 1.2	24.4 ± 0.8	34.6 ± 0.9	21.8 ± 0.7	27.1 ± 1.0	31.7 ± 0.7
	**Percent Consumer**	**Nutrient Density**							
		Percentage of energy from nutrient ± SE *			Nutrient/1000 KJ ± SE *				
	%	%	%	%	mg	mg	mg	μg retinol equivalents	mg
**Breakfast**	81.1	19.8 ± 1.1	9.6 ± 0.3	14.3 ± 0.7	277.4 ± 9.4	159.0 ± 5.0	2.1 ± 0.1	83.2 ± 5.9	44.1 ± 0.9
**Lunch**	87.6	32.4 ± 1.3	11.1 ± 0.3	9.8 ± 0.5	414.8 ± 9.1	71.7 ± 2.5	1.1 ± 0.0	70.4 ± 6.0	29.5 ± 0.5
**Dinner**	94.1	27.6 ± 1.3	12.5 ± 0.3	7.8 ± 0.4	330.3 ± 9.8	66.0 ± 2.5	1.2 ± 0.0	118.9 ± 8.8	30.2 ± 0.4
**Other Combined**	99.2	57.0 ± 1.3	13.1 ± 0.3	24.0 ± 0.8	210.1 ± 6.0	109.7 ± 3.8	0.8 ± 0.0	61.2 ± 4.3	39.5 ± 1.5
**TOTAL**	-	38.4 ± 0.8	12.8 ± 0.2	14.0 ± 0.3	309.3 ± 4.6	93.4 ± 1.7	1.2 ± 0.0	78.0 ± 2.5	32.8 ± 0.3
		Nutrient Density Index (% of daily nutrient/% of daily energy) ± SE *							
**Breakfast**		0.6 ± 0.1	0.8 ± 0.0	1.2 ± 0.1	0.9 ± 0.0	1.7 ± 0.0	1.6 ± 0.0	1.2 ± 0.1	1.3 ± 0.0
**Lunch**		0.9 ± 0.0	0.9 ± 0.0	0.7 ± 0.0	1.4 ± 0.0	0.8 ± 0.0	1.0 ± 0.0	0.9 ± 0.1	0.9 ± 0.0
**Dinner**		0.7 ± 0.0	1.0 ± 0.0	0.6 ± 0.0	1.0 ± 0.0	0.8 ± 0.0	1.1 ± 0.0	1.3 ± 0.0	0.9 ± 0.0
**Other Combined**		1.8 ± 0.1	1.0 ± 0.0	1.8 ± 0.1	0.7 ± 0.0	1.2 ± 0.0	0.7 ± 0.0	0.9 ± 0.0	1.2 ± 0.0

^†^ Percent contribution represents the mean of the percent contribution of each respondent. ^1^ Breakfast is defined as the self-defined eating occasion ‘breakfast’; Lunch includes ‘lunch’ and ‘brunch’; Dinner is ‘dinner’ only; and Other Combined combined the total of ‘morning tea’, ‘afternoon tea’, ‘snack’, ‘supper’, ‘beverage/drink’, ‘extended consumption’, and ‘other’. * SE—standard error.

**Table 2:** Top five sub-major food groups ranked by the contribution to total daily energy and nutrient intakes.

Nutrient	Top Five Sub-Major Food Groups	Contribution to Total Daily Nutrient Intake (% ^†^ ± 95% MoE *)	Contribution to Total Daily Nutrient Intake by Eating Occasion ^1^ (% ^†^ ± 95% MoE *)			
			Breakfast	Lunch	Dinner	Other Combined
**Energy**	Regular breads, and bread rolls (plain/unfilled/untopped varieties)	6.8 ± 1.0	1.9 ± 0.3	3.1 ± 0.6	0.8 ± 0.2	1.1 ± 0.3
	Potatoes	4.8 ± 1.2	0.1 ± 0.1	1.6 ± 0.8	2.6 ± 0.7	0.4 ± 0.2
	Dairy milk (cow, sheep and goat)	4.3 ± 0.6	2.2 ± 0.4	0.1 ± 0.1	0.2 ± 0.1	1.8 ± 0.4
	Burgers	4.2 ± 1.0	0.1 ± 0.1	1.7 ± 0.6	1.9 ± 0.7	0.6 ± 0.3
	Cakes, muffins, scones, cake-type desserts	3.7 ± 1.0	0.2 ± 0.2	0.6 ± 0.5	0.3 ± 0.2	2.5 ± 0.9
	**Sum of top sources ^2^**	23.9	4.5	7.2	5.8	6.4
**Discretionary energy**	Soft drinks, and flavoured mineral waters	8.9 ± 1.5	0.1 ± 0.2	2.2 ± 0.7	2.5 ± 0.6	4.0 ± 0.9
	Cakes, muffins, scones, cake-type desserts	8.4 ± 2.2	0.6 ± 0.5	1.6 ± 1.2	0.8 ± 0.5	5.5 ± 1.7
	Potatoes	8.4 ± 2.3	0.3 ± 0.3	3.4 ± 2.0	3.8 ± 1.6	0.9 ± 0.5
	Pastries	7.4 ± 1.9	0.4 ± 0.5	3.8 ± 1.5	1.7 ± 0.8	1.5 ± 0.7
	Chocolate and chocolate-based confectionery	5.4 ± 1.5	0.0 ± 0.0	0.3 ± 0.3	0.2 ± 0.1	4.9 ± 1.4
	**Sum of top sources ^2^**	38.6	1.4	11.4	9.0	16.8
**Saturated fat**	Dairy milk (cow, sheep and goat)	8.2 ± 1.2	4.2 ± 0.7	0.2 ± 0.1	0.3 ± 0.2	3.4 ± 0.9
	Cheese	6.4 ± 1.1	0.3 ± 0.2	2.5 ± 0.6	1.5 ± 0.6	2.1 ± 0.9
	Chocolate and chocolate-based confectionery	5.0 ± 1.4	0.0 ± 0.0	0.3 ± 0.3	0.2 ± 0.1	4.5 ± 1.3
	Pastries	4.9 ± 1.3	0.2 ± 0.3	2.4 ± 0.9	1.2 ± 0.6	1.0 ± 0.5
	Cakes, muffins, scones, cake-type desserts	4.8 ± 1.4	0.2 ± 0.2	0.7 ± 0.6	0.6 ± 0.4	3.3 ± 1.3
	**Sum of top sources ^2^**	29.3	5.0	6.1	3.8	14.4
**Free sugars**	Soft drinks, and flavoured mineral waters	25.3 ± 4.2	0.4 ± 0.4	6.3 ± 2.0	7.2 ± 1.7	11.4 ± 2.7
	Fruit and vegetable juices, and drinks	13.7 ± 2.9	4.2 ± 1.4	2.5 ± 1.2	3.2 ± 1.2	3.7 ± 1.4
	Cakes, muffins, scones, cake-type desserts	6.9 ± 1.6	0.3 ± 0.3	1.3 ± 0.6	0.7 ± 0.5	4.6 ± 1.5
	Sugar, honey and syrups	6.4 ± 1.5	3.4 ± 1.2	0.4 ± 0.4	0.3 ± 0.3	2.2 ± 1.0
	Chocolate and chocolate-based confectionery	5.1 ± 1.4	0.0 ± 0.0	0.4 ± 0.5	0.2 ± 0.2	4.5 ± 1.3
	**Sum of top sources ^2^**	53.9	5.7	10.6	10.8	26.9
**Sodium**	Regular breads, and bread rolls (plain/unfilled/untopped varieties)	10.7 ± 1.5	2.9 ± 0.5	4.9 ± 1.0	1.2 ± 0.3	1.7 ± 0.5
	Burgers	6.9 ± 1.7	0.1 ± 0.2	2.8 ± 1.0	3.1 ± 1.2	0.9 ± 0.5
	Processed meat	6.4 ± 1.4	1.9 ± 0.9	2.2 ± 0.6	1.6 ± 0.8	0.7 ± 0.5
	Pizza	4.6 ± 1.3	0.2 ± 0.2	0.8 ± 0.5	3.5 ± 1.2	0.1 ± 0.1
	Pastries	4.2 ± 1.2	0.3 ± 0.4	2.4 ± 1.0	0.7 ± 0.4	0.8 ± 0.4
	**Sum of top sources ^2^**	32.7	5.3	13.0	10.2	4.2
**Calcium**	Dairy milk (cow, sheep and goat)	21.0 ± 2.5	10.8 ± 1.5	0.5 ± 0.3	1.0 ± 0.6	8.7 ± 1.9
	Cheese	9.7 ± 1.8	0.4 ± 0.3	3.8 ± 1.0	2.4 ± 0.9	3.0 ± 1.4
	Flavoured milks and milkshakes	6.4 ± 2.2	1.2 ± 0.8	0.9 ± 0.6	0.6 ± 0.7	3.7 ± 2.0
	Regular breads, and bread rolls (plain/unfilled/untopped varieties)	5.2 ± 0.7	1.3 ± 0.2	2.5 ± 0.5	0.6 ± 0.2	0.8 ± 0.2
	Pizza	4.4 ± 1.4	0.2 ± 0.2	0.7 ± 0.5	3.4 ± 1.4	0.1 ± 0.1
	**Sum of top sources ^2^**	46.8	13.8	8.5	8.0	16.4
**Iron**	Breakfast cereals, ready to eat	17.8 ± 3.2	15.6 ± 2.6	0.3 ± 0.3	0.0 ± 0.1	1.8 ± 1.2
	Regular breads, and bread rolls (plain/unfilled/untopped varieties)	9.0 ± 1.2	2.6 ± 0.5	4.0 ± 0.8	0.9 ± 0.3	1.4 ± 0.4
	Beef, sheep and pork, unprocessed	6.0 ± 1.3	0.0 ± 0.0	1.1 ± 0.6	4.6 ± 1.1	0.3 ± 0.3
	Burgers	4.4 ± 1.1	0.1 ± 0.1	1.7 ± 0.7	2.0 ± 0.7	0.6 ± 0.3
	Pasta and dumplings	4.0 ± 1.0	0.0 ± 0.0	0.9 ± 0.5	2.6 ± 0.7	0.4 ± 0.2
	**Sum of top sources ^2^**	41.1	18.3	8.1	10.2	4.4
**Vitamin A**	Carrot and similar root vegetables	18.6 ± 7.2	0.0 ± 0.0	3.7 ± 3.8	12.1 ± 6.3	2.8 ± 2.6
	Dairy milk (cow, sheep and goat)	8.8 ± 1.2	4.5 ± 0.7	0.2 ± 0.2	0.4 ± 0.2	3.6 ± 1.0
	Frozen milk products	5.4 ± 1.4	0.0 ± N/A	0.5 ± 0.6	2.0 ± 1.0	3.0 ± 1.1
	Soup, homemade from basic ingredients	4.0 ± 3.1	0.5 ± 1.0	1.0 ± 0.9	2.4 ± 3.0	0.1 ± 0.1
	Flavoured milks and milkshakes	3.9 ± 1.4	0.6 ± 0.5	0.5 ± 0.4	0.3 ± 0.3	2.5 ± 1.4
	**Sum of top sources ^2^**	40.7	5.6	5.9	17.2	12.0
**Magnesium**	Regular breads, and bread rolls (plain/unfilled/untopped varieties)	7.6 ± 1.0	2.2 ± 0.4	3.5 ± 0.6	0.8 ± 0.3	1.2 ± 0.3
	Breakfast cereals, ready to eat	6.3 ± 1.3	5.7 ± 1.1	0.1 ± 0.1	0.0 ± 0.0	0.5 ± 0.4
	Waters, municipal and bottled, unflavoured	6.2 ± 0.7	0.2 ± 0.1	0.3 ± 0.1	0.2 ± 0.1	5.5 ± 0.7
	Dairy milk (cow, sheep and goat)	5.8 ± 0.7	3.0 ± 0.4	0.1 ± 0.1	0.3 ± 0.2	2.4 ± 0.5
	Potatoes	4.8 ± 1.2	0.1 ± 0.1	1.5 ± 0.8	2.8 ± 0.6	0.4 ± 0.2
	**Sum of top sources ^2^**	30.6	11.1	5.4	4.1	10.0

^†^ Represents the proportion of total nutrient intake from all respondents that came from each sub-major food group. ^1^ Breakfast is defined as the self-defined eating occasion ‘breakfast’; Lunch includes ‘lunch’ and ‘brunch’; Dinner is ‘dinner’ only; and Other Combined combined the total of ‘morning tea’, ‘afternoon tea’, ‘snack’, ‘supper’, ‘beverage/drink’, ‘extended consumption’, and ‘other’. ^2^ The proportion of the total nutrient intake that came from the top five food groups combined. * MoE—margin of error.

## Supplementary Materials

The following are available online at www.mdpi.com/2072-6643/9/6/608/s1, Supplementary Table S1: Food grouping definitions for the 52 foods groups, Supplementary Table S2: Prevalence of consumers of each food group at each eating occasion, Supplementary Table S3: Mean energy intake (kJ) per capita for each food group (and grams for selected food groups) by eating occasion.
