# Supplementary material for: Identifying Eating Occasion-Based Opportunities to Improve the Overall Diets of Australian Adolescents
Source: Nutrients. 2017 Jun 14;9(6):608. doi: 10.3390/nu9060608 (PMC5490587; doi:10.3390/nu9060608)
Supplement: Supplementary file 1 [file nutrients-09-00608-s001.zip › nutrients-193099-supplementary.pdf]

Supplemental Table S1: Food grouping definitions for the 52 foods groups.

Name	Description[1]	8-Digit Food Codes Included[2]
Tea and coffee	Tea and coffee without milk, coffee beverages including milk (cappuccino or flat white)	11101001-11209005
Fruit and vegetable juice	Commercial and non-commercial juices	11301001-11306003
Sweetened beverages	Fruit drinks, cordials, soft drinks, flavored mineral waters, sports drinks, and energy drinks	11307001-11604003
Water	Unflavored water	11701001-11703001
Liquid breakfast, fortified beverages (like Milo)	Milo prepared with milk, unfortified beverage flavourings prepared with milk, beverage bases unmixed, and breakfast cereal beverages	11801001-11806002
Rice, grains and flours	Oats, rice, quinoa and couscous without additions	12101001-12104002
Breads and rolls	Bagels, bread, English muffins, and flat breads	12201001-12305006;<br>12307001-12307004
Desserts	Sweet buns, sweet biscuits, cakes, cupcakes, banana breads, cake-style muffins with chocolate, pudding, pie, danishes, éclairs, doughnuts, ice cream, toppings, pavlova, and ice confections	12305007-12305010;<br>12306001-12306009;<br>13101001-13107001;<br>13301001-13303077;<br>13304011-13304014;<br>13304018-13304019;<br>13305001-13306029;<br>13308003;<br>13309001-13309004;<br>13402001-13403021;<br>13605001-13605011;<br>16901001-16901007;<br>19501001-19508011;<br>19601001-19602007;<br>19701001-19702007;<br>27103001-27103006;<br>27301001-27304015
Pasta and noodles	Pasta and noodles without additions. Filled pasta (meat, spinach, cheese, vegetables)	12401001-12404004
Breakfast cereal	Ready-to-eat breakfast cereals and muesli	12501001-12516003
Hot porridge	Porridge with and without (milk, sugar, honey and dried fruit)	12601001-12602003
Savory biscuits	Crackers, wafers, rice cakes, and crispbreads,	13201001-13205003
Muffins and scones	Un-iced cake-style muffins and scones	13304001-13304010;<br>13304015-13304017;<br>13304020-13304021;<br>13307001-13308002
Savory pastries	Croissants, quiche, pastries filled with vegetables or meat, meat pies, sausage rolls, samosas, and spring rolls	13401001-13401024;<br>13404001-13406017
Pizza	Pizza with and without toppings	13501001-13502022
Sandwiches	Sandwiches, filled bread rolls, hot dogs, kebab wrap, and wraps	13503001-13504007
Burgers	Bacon burgers, chicken burgers, fish burgers, hamburgers, English muffins with egg, cheese and or beef/bacon, steak sandwiches, vegetable or lentil burgers	13505001-13506006
Tacos	Tacos, nachos, Mexican-style wraps	13507001-13508012
Pasta and dumpling dishes	Lasagna, macaroni and cheese, pasta with sauce, stir-fry with rice noodles, steamed buns	13509001-13510008;<br>13513001-13513007;<br>13515001-13515006
Rice dishes and sushi	Paella, rice with (meat, eggs and/or vegetables), risotto, sushi with (fish, chicken, prawns, or vegetables)	13511001-13511030;<br>13514001-13514023
Batter-based products	Crepes, pancakes, pikelet, waffles, fritters, Yorkshire pudding, crumpets without additions.	13601001-13604010;<br>13606001-13606003
Fats and oils	Butter, margarine, and oils	14101001-14602008
Fish and fish mixed dish	Fish (baked, boiled, roasted, smoked, battered, and fried). Crabs, prawns, scallops, squid, and canned fish. Fish casseroles, and seafood stir-fry.	15101001-15604005

Fruit	Raw fruits and fruit salads	16101001-16101020; 16103001-16103011; 16105001-16105002; 16201001-16201012; 16301001-16303005; 16401001-16401011; 16403001-16403006; 16501001-16504012; 16601001-16701032
Commercial fruit, dried fruit	Canned fruits (drained, with juice, or syrup), and dried fruits	16102001; 16104001-16104013; 16202001-16202010; 16304001-16304004; 16402001-16402013; 16404001-16404018; 16505001-16505010; 16702001-16702015; 16801001-16804004
Eggs and egg dishes	Eggs, eggs with additions (bacon, cheese). Frittatas, omelettes, and eggs benedict	17101001- 17202003
Meat	Beef, lamb, pork, veal, kangaroo, chicken, duck, sausages (beef, pork, chicken)	18101001-18503009
Processed meat	Bacon, ham, prosciutto, salami, corned beef, turkey deli-sliced, spam, and beef jerky	18601001-18607001
Mixed Dish with meat	Casseroles (beef, pork, chicken), curries (beef, pork, chicken), beef Bolognese sauce, stir-fry (beef, pork, chicken), coated chicken, chicken nuggets, and meatloaf	18701001-18903054
Dairy milk	Fluid milk, condensed milk, and milk powder	19101001-19109004
Yoghurt	Plain and flavored yogurts	19201001-19212002
Cream	Cream, sour cream, and whipped cream	19301001-19306004
Cheese	Cheese	19401001-19408004
Flavored milk	Iced coffee, milk with flavoring, milkshakes, thickshakes, and fruit smoothies with milk	19801001-19806007
Dairy milk substitutes	Soy beverages and rice beverages including smoothies and milkshakes, and soy based yogurts	20101001-20502003
Meat substitutes and dishes	Vegetarian sausages, tofu, tempeh, veggie burgers, stir-fry with tofu	20601001-20701004
Soup	Homemade, canned, and soups prepared from dry mixes	21101001-21602002
Seeds and nuts	Chia seeds, nuts (peanuts, almonds, pistachio, pine), peanut butter, satay sauce, and coconut milk	22101001-22205019
Savory sauces	Gravies, barbecue sauce, tomato sauce, soy sauce, curry paste, tabasco sauce, sweet and sour sauce, and cheese sauce	23101001-23110002
Dressings, stuffings, dips	Chutney, pickled vegetables, salad dressing, mayonnaise, tartare sauce, balsamic vinegar, dairy-based dips, guacamole, and hummus	23201001-23202010; 23301001-23305002; 23401001; 23501001-23504003
Potatoes	Baked, boiled, fried, grilled, or BBQ'd potatoes. Fries, hashbrowns, and wedges. Baked potatoes with additions (cream, cheese, meat, sour cream)	24101001-24103021
Vegetables (non-potato)	Vegetables (including sweet potato), vegetables cook with and without fat	24201001-24803034
Vegetable dishes	Vegetable casseroles, vegetable curries with (rice, noodles), vegetable stir-fry, vegetable salads with additions (cheese, fruit, nuts meat, eggs), coleslaw, and potato salad	24901001-24905023
Legumes and legume dishes	Beans, lentils, baked beans plain and in (sauces, curry, salads and casseroles)	25101001-25202016
Savory snacks	Potato crisps, potato straws, corn chips, popcorn with additions (salt, butter), extruded snacks, snack mixes, and pretzels	26101001-26401016
Sugar, honey, syrups	Sugar, honey, and maple syrups	27101001-27102005
Jams and spreads	Fruit jams, chocolate/hazelnut spread	27201001-27205003
Confectionary	Chocolate, bars, licorice, honeycomb, lollies, marshmallows, and chewing gum	28101001-28104001; 28401001-28405003
Muesli and cereal bars	Fruit bars, fruit leathers, muesli bars with and without coatings or chocolate chips	28201001-28202008; 28301001-28305004
Alcohol	Beer, wine, spirits, and mixed drinks	29101001-29505003
Formula dietary foods	Meal replacements (powders, beverages, bars). Sport and protein beverages and powders. Supplementary and medical foods (beverages and powders)	30101001-30107006
Miscellaneous	Yeast spreads, intense-sweeteners (aspartame, sucralose, saccharin), salt, dried spices, stock (dry powder, cube, or liquid)	31101001-31503011

[1]The foods listed in the description are not comprehensive and serve to provide examples of foods and beverages included in a given food group. A comprehensive list for each food group is provided with the 8-digit food codes.
[2]Available 8-digit food codes were allocated to the 52 food groups; however, the 14-18 year olds in the sample did not report consuming all of the 8-digit food codes available.

Supplementary Table S2: Prevalence of consumers of each food group at each eating occasion[1]

Food Groups	OVERALL		Breakfast		Lunch		Dinner		Other combined	
	% consumers	Rank	% consumers	Rank	% consumers	Rank	% consumers	Rank	% consumers	Rank
Water	87.8%	1	7.0%	10	10.7%	10	9.8%	12	78.7%	1
Bread and rolls	64.9%	2	28.2%	3	34.4%	1	13.0%	7	15.9%	8
Sweetened beverages	60.8%	3	6.7%	11	21.5%	2	23.4%	4	31.0%	4
Dairy milk	56.8%	4	39.8%	1	3.3%	25	5.0%	20	23.6%	6
Desserts	48.7%	5	0.4%	37	6.8%	16	10.8%	10	40.6%	2
Fruit	46.6%	6	6.4%	13	11.6%	9	3.4%	24	35.9%	3
Vegetables (non-potato)	42.5%	7	2.0%	23	17.1%	3	26.5%	2	7.8%	16
Fats and oils	38.0%	8	18.9%	5	15.0%	4	7.1%	16	8.5%	15
Meat	37.5%	9	1.1%	28	11.7%	8	27.1%	1	4.1%	22
Potatoes	36.0%	10	1.2%	26	9.6%	11	23.7%	3	4.8%	20
Cheese	31.8%	11	2.3%	22	14.7%	5	9.2%	13	9.6%	13
Breakfast cereal	30.9%	12	29.1%	2	0.8%	43	0.3%	45	2.2%	33
Confectionary	29.5%	13	0.5%	35	2.3%	33	1.5%	34	26.0%	5
Sugar, honey and syrups	29.1%	14	19.6%	4	2.9%	29	0.9%	38	11.2%	11
Savoury sauces	28.9%	15	2.7%	19	12.7%	7	13.2%	6	5.6%	19
Mixed dishes with meat	28.4%	16	0.4%	38	7.4%	12	21.1%	5	0.9%	42
Tea and coffee	26.5%	17	12.7%	6	2.7%	31	1.6%	33	14.5%	9
Processed meat	25.5%	18	6.2%	14	14.1%	6	4.6%	21	3.2%	27
Fruit and vegetable juices	22.2%	19	9.4%	8	3.7%	21	5.4%	19	7.1%	17
Savory snacks	21.7%	20	0.3%	42	3.6%	22	1.3%	35	17.4%	7
Dressing, stuffing, dips	17.2%	21	0.0%	48	7.1%	14	7.6%	14	3.9%	23
Burgers	16.6%	22	0.3%	44	7.2%	13	7.3%	15	2.8%	28
Savoury biscuits	16.0%	23	0.7%	32	3.2%	26	0.2%	46	12.3%	10
Miscellaneous	15.9%	24	7.2%	9	4.9%	17	2.9%	27	2.7%	30
Eggs and egg dishes	15.6%	25	9.9%	7	1.9%	36	3.3%	25	0.6%	46
Pasta and dumpling dishes	15.0%	26	0.3%	43	3.9%	20	9.9%	11	2.2%	32
Rice, grains and flours	14.8%	27	1.9%	24	3.3%	24	11.3%	8	1.0%	40
Liquid breakfast meals and fortified beverages	14.7%	28	6.5%	12	0.1%	49	0.1%	47	8.7%	14
Muesli and cereal bars	14.2%	29	0.2%	45	2.8%	30	0.4%	43	11.0%	12
Vegetable dishes	13.3%	30	0.0%	49	2.4%	32	11.0%	9	0.5%	49
Savoury pastries	12.8%	31	0.9%	29	7.0%	15	3.0%	26	3.3%	26
Fish and fish mixed dishes	12.4%	32	0.6%	34	4.1%	19	6.8%	17	1.8%	34
Flavoured milk	11.8%	33	2.6%	20	2.1%	35	0.9%	37	6.4%	18
Nuts and seeds	11.7%	34	5.0%	15	3.0%	28	0.4%	44	3.4%	25
Pasta and noodles	11.4%	35	0.4%	39	4.6%	18	4.4%	22	2.6%	31
Pizza	8.6%	36	0.4%	40	1.6%	38	6.2%	18	0.5%	48
Yoghurt	8.3%	37	2.3%	21	1.2%	40	0.8%	39	4.2%	21
Jams and spreads	8.1%	38	2.9%	18	1.8%	37	0.6%	40	3.5%	24
Rice dishes and sushi	7.5%	39	0.0%	47	3.5%	23	3.9%	23	1.0%	41
Soup	6.3%	40	0.6%	33	2.1%	34	2.7%	29	0.8%	43
Sandwiches	6.1%	41	0.4%	36	3.0%	27	1.7%	32	1.2%	39
Batter-based products	4.8%	42	3.0%	17	0.0%	50	0.5%	42	1.2%	38
Legumes and legume dishes	4.4%	43	1.4%	25	0.8%	44	2.9%	28	0.2%	50

Alcohol	4.2%	44	0.0%	49	0.2%	46	1.3%	36	2.7%	29
Tacos	4.2%	45	0.0%	49	1.2%	41	2.4%	30	0.7%	45
Cream	3.5%	46	0.1%	46	0.8%	42	1.8%	31	0.8%	44
Commercial fruit, dried fruit	3.4%	47	0.3%	41	1.4%	39	0.5%	41	1.2%	37
Hot porridge	3.1%	48	3.0%	16	0.0%	51	0.0%	50	0.1%	52
Formula dietary foods	2.4%	49	0.8%	30	0.1%	48	0.1%	49	1.4%	36
Muffins and scones	2.4%	50	0.7%	31	0.5%	45	0.0%	50	1.4%	35
Dairy milk substitutes	1.7%	51	1.1%	27	0.1%	47	0.0%	50	0.5%	47
Meat substitutes and dishes	0.2%	52	0.0%	49	0.0%	51	0.1%	48	0.1%	51

[1]Breakfast is defined as the self-defined eating occasion 'breakfast'; Lunch includes 'lunch' and 'brunch'; Dinner is 'dinner' only; and other combined the total of 'morning tea', 'afternoon tea', 'snack', 'supper', 'beverage/drink', 'extended consumption', and 'other'.

Supplementary Table S3: Mean energy intake (kJ) per capita for each food group (and grams for selected food groups) by eating occasion.

Food Groups	Total		Breakfast		Lunch		Dinner		Other EO combined	
	Grams (mean ± SE*)	Rank	Grams (mean ± SE*)	Rank	Grams (mean ± SE*)	Rank	Grams (mean ± SE*)	Rank	Grams (mean ± SE*)	Rank
Water	1123.7 ± 32.5	1	48.6 ± 8.9	1	45.6 ± 6.3	1	46.2 ± 6.0	1	983.1 ± 33.3	1
Fruit	103.0 ± 5.9	2	9.7 ± 1.5	4	19.8 ± 2.4	2	6.0 ± 1.5	5	67.4 ± 4.9	2
Fruit and vegetable juice	88.2 ± 7.3	3	30.8 ± 3.7	2	12.2 ± 2.4	5	20.2 ± 3.9	4	25.1 ± 3.6	4
Tea and coffee	85.7 ± 6.4	4	27.6 ± 2.7	3	5.3 ± 1.2	6	4.9 ± 1.7	6	47.9 ± 5.0	3
Potatoes	56.2 ± 4.2	5	1.2 ± 0.4	6	16.0 ± 2.7	3	34.9 ± 3.1	2	4.2 ± 0.8	6
Vegetables (non-potato)	51.3 ± 3.4	6	1.4 ± 0.5	5	13.1 ± 1.5	4	31.3 ± 2.7	3	5.5 ± 1.0	5
Commercial fruit, dried fruit	4.5 ± 1.1	7	0.4 ± 0.3	7	2.5 ± 0.9	7	0.4 ± 0.3	7	1.2 ± 0.6	7
**Food Groups**	**kJ (mean ± SE*)**	**Rank**	**kJ (mean ± SE*)**	**Rank**	**kJ (mean ± SE*)**	**Rank**	**kJ (mean ± SE*)**	**Rank**	**kJ (mean ± SE*)**	**Rank**
Desserts	798.8 ± 45.8	1	8.6 ± 5.9	25	85.9 ± 14.0	8	142.7 ± 19.4	7	561.6 ± 37.7	1
Bread, rolls	741.0 ± 29.1	2	197.7 ± 13.8	3	309.7 ± 18.4	1	92.7 ± 10.0	11	140.6 ± 13.1	8
Meat	543.5 ± 37.1	3	7.9 ± 3.8	27	120.9 ± 18.0	7	386.0 ± 30.5	1	28.8 ± 5.8	23
Sweetened beverages	516.2 ± 26.4	4	32.7 ± 5.1	10	123.4 ± 10.5	6	127.3 ± 10.3	9	232.7 ± 17.3	2
Mixed dish with meat	498.9 ± 35.6	5	3.4 ± 2.1	37	138.4 ± 22.0	5	347.8 ± 28.5	2	9.3 ± 4.8	37
Potatoes	440.5 ± 34.3	6	12.2 ± 4.1	21	145.3 ± 23.7	3	242.7 ± 23.7	3	40.4 ± 7.6	17
Dairy milk	397.7 ± 21.9	7	204.0 ± 12.5	2	10.4 ± 3.1	33	18.0 ± 4.2	26	165.2 ± 15.3	5
Burgers	386.8 ± 36.0	8	6.7 ± 5.1	30	157.5 ± 22.7	2	169.9 ± 23.7	6	52.7 ± 12.1	12
Pasta and dumpling dishes	316.7 ± 34.3	9	0.4 ± 0.3	46	77.5 ± 16.6	9	205.6 ± 26.1	4	33.2 ± 9.4	18
Breakfast cereal	312.2 ± 21.9	10	277.6 ± 18.9	1	5.4 ± 2.5	39	0.8 ± 0.5	45	28.4 ± 8.2	24
Savoury pastries	253.8 ± 29.6	11	13.7 ± 6.2	19	140.6 ± 20.4	4	56.8 ± 14.4	16	42.7 ± 9.9	16
Confectionary	252.9 ± 23.7	12	1.7 ± 1.1	41	14.8 ± 4.9	31	10.8 ± 4.0	32	225.5 ± 23.0	3
Pizza	251.4 ± 34.6	13	10.4 ± 6.5	23	41.5 ± 13.5	16	194.4 ± 31.6	5	5.0 ± 2.8	43
Fruit	236.6 ± 12.4	14	21.1 ± 3.4	15	46.7 ± 5.9	13	13.3 ± 3.5	30	155.5 ± 9.9	6
Savory snacks	215.1 ± 23.1	15	2.0 ± 1.7	40	22.0 ± 4.6	22	12.2 ± 5.0	31	178.9 ± 22.1	4
Rice, grains and flours	210.0 ± 22.8	16	16.6 ± 5.2	17	42.6 ± 9.9	15	131.3 ± 14.8	8	19.4 ± 8.0	30
Pasta and noodles	202.0 ± 28.0	17	5.4 ± 3.2	32	62.8 ± 11.8	11	103.8 ± 24.3	10	30.0 ± 7.2	22
Flavoured milk	195.5 ± 22.6	18	31.7 ± 7.5	12	27.5 ± 7.7	19	16.7 ± 6.9	27	119.6 ± 18.9	9
Savoury biscuits	170.5 ± 19.9	19	2.1 ± 1.0	39	23.2 ± 5.8	21	2.8 ± 2.1	36	142.4 ± 19.1	7
Cheese	170.3 ± 12.4	20	8.6 ± 2.5	24	65.6 ± 6.6	10	39.6 ± 6.4	19	56.3 ± 8.4	11
Sandwiches	144.5 ± 23.4	21	11.6 ± 7.2	22	60.7 ± 14.5	12	40.6 ± 12.4	18	31.6 ± 10.8	21
Rice dishes and sushi	136.2 ± 20.4	22	1.4 ± 2.2	43	43.0 ± 8.9	14	80.4 ± 16.2	12	11.5 ± 4.9	35
Tacos	123.1 ± 24.0	23	0.0 ± N/A	48	21.2 ± 7.6	25	78.2 ± 20.4	13	23.6 ± 10.7	28

Fruit and vegetable juice	114.7 ± 9.6	24	39.6 ± 4.8	8	15.8 ± 3.1	30	27.2 ± 5.6	23	32.0 ± 4.6	20
Fats and oils	112.0 ± 9.3	25	41.7 ± 4.3	7	28.2 ± 3.2	18	16.2 ± 3.1	28	26.0 ± 5.9	27
Processed meat	111.6 ± 10.7	26	35.2 ± 5.6	9	35.7 ± 4.5	17	26.2 ± 6.3	24	14.5 ± 4.7	33
Fish and fish mixed dish	101.2 ± 13.9	27	1.5 ± 0.9	42	21.2 ± 5.4	26	67.6 ± 11.9	14	10.9 ± 3.4	36
Vegetables (non-potato)	91.9 ± 7.8	28	4.7 ± 2.0	34	21.8 ± 4.3	23	57.8 ± 6.0	15	7.7 ± 1.6	39
Muesli and cereal bars	88.5 ± 9.3	29	4.2 ± 4.3	35	16.4 ± 3.9	29	2.1 ± 1.2	39	65.8 ± 7.4	10
Muffins and scones	87.4 ± 24.5	30	19.5 ± 8.5	16	21.4 ± 11.5	24	0.0 ± N/A	49	46.5 ± 17.5	14
Eggs and egg dishes	78.1 ± 8.3	31	50.8 ± 7.0	5	7.5 ± 2.2	37	15.4 ± 3.4	29	4.3 ± 2.4	45
Sugar,honey, syrups	77.3 ± 6.5	32	44.6 ± 5.1	6	4.9 ± 1.1	40	1.3 ± 0.6	41	26.6 ± 3.3	25
Liquid breakfast, fortified beverages (like Milo)	75.9 ± 8.4	33	32.3 ± 5.4	11	0.2 ± 0.2	49	0.2 ± 0.4	48	43.2 ± 6.3	15
Dressing, stuffing, dips	72.0 ± 9.1	34	0.0 ± 0.1	47	26.6 ± 4.5	20	28.7 ± 6.4	21	16.6 ± 4.6	32
Seeds and nuts	68.5 ± 11.4	35	23.1 ± 4.7	13	10.0 ± 2.5	34	2.7 ± 5.6	37	32.6 ± 8.3	19
Vegetable dishes	66.0 ± 13.9	36	0.0 ± N/A	48	8.2 ± 3.5	36	53.8 ± 12.3	17	4.0 ± 2.7	46
Legumes and legume dishes	64.4 ± 23.1	37	14.1 ± 5.1	18	19.9 ± 11.1	27	27.6 ± 8.4	22	2.8 ± 3.6	47
Alcohol	60.6 ± 15.9	38	0.0 ± N/A	48	0.7 ± 0.6	45	7.8 ± 2.7	34	52.1 ± 15.7	13
Savoury sauces	55.6 ± 6.7	39	2.2 ± 0.6	38	17.2 ± 2.9	28	31.6 ± 5.7	20	4.7 ± 1.1	44
Hot porridge	55.2 ± 13.6	40	54.2 ± 13.6	4	0.0 ± N/A	50	0.0 ± N/A	49	1.0 ± 1.6	49
Yoghurt	48.7 ± 7.3	41	12.3 ± 3.4	20	8.4 ± 3.5	35	1.5 ± 0.6	40	26.5 ± 5.0	26
Soup	45.9 ± 8.0	42	5.6 ± 2.6	31	14.6 ± 4.0	32	18.6 ± 5.6	25	7.1 ± 3.3	40
Jams and spreads	34.2 ± 7.1	43	8.3 ± 2.0	26	4.1 ± 1.5	41	2.7 ± 1.3	38	19.1 ± 6.5	31
Tea and coffee	31.2 ± 4.7	44	7.6 ± 1.5	28	1.2 ± 0.8	44	0.6 ± 0.3	46	21.8 ± 4.2	29
Batter-based products	30.2 ± 6.2	45	21.7 ± 5.7	14	0.4 ± 0.6	48	3.1 ± 1.7	35	5.1 ± 1.7	42
Cream	21.3 ± 6.1	46	0.6 ± 0.6	45	3.8 ± 1.7	42	8.5 ± 3.3	33	8.4 ± 4.8	38
Formula dietary foods	19.1 ± 5.6	47	5.2 ± 2.5	33	0.4 ± 0.6	47	1.0 ± 1.2	43	11.9 ± 4.9	34
Commercial fruit, dried fruit	14.1 ± 3.2	48	1.3 ± 0.8	44	6.6 ± 2.3	38	0.9 ± 0.7	44	5.4 ± 2.0	41
Dairy milk substitutes	9.6 ± 3.7	49	7.2 ± 3.4	29	0.5 ± 0.5	46	0.0 ± N/A	49	2.0 ± 1.1	48
Miscellaneous	6.4 ± 0.8	50	3.4 ± 0.6	36	2.0 ± 0.4	43	0.4 ± 0.1	47	0.6 ± 0.2	51
Meat substitutes and dishes	2.1 ± 1.6	51	0.0 ± N/A	48	0.0 ± N/A	50	1.2 ± 1.4	42	0.9 ± 0.9	50
Water	0.4 ± 0.5	52	0.0 ± 0.0	48	0.0 ± 0.0	50	0.0 ± 0.0	49	0.4 ± 0.5	52

[1]Breakfast is defined as the self-defined eating occasion 'breakfast'; Lunch includes 'lunch' and 'brunch'; Dinner is 'dinner' only; and other combined the total of 'morning tea', 'afternoon tea', 'snack', 'supper', 'beverage/drink', 'extended consumption', and 'other'. [*]SE = standard error.
